# Hemogram-Derived Inflammatory Markers in Cats with Chronic Kidney Disease

**DOI:** 10.3390/ani14121813

**Published:** 2024-06-18

**Authors:** Martina Krofič Žel, Alenka Nemec Svete, Nataša Tozon, Darja Pavlin

**Affiliations:** Small Animal Clinic, Veterinary Faculty, University of Ljubljana, Gerbičeva 60, 1000 Ljubljana, Slovenia; martina.kroficzel@vf.uni-lj.si (M.K.Ž.); alenka.nemecsvete@vf.uni-lj.si (A.N.S.); natasa.tozon@vf.uni-lj.si (N.T.)

**Keywords:** chronic kidney disease, cats, hemogram-derived inflammatory markers, inflammation, neutrophil-to-lymphocyte ratio, monocyte-to-lymphocyte ratio, platelet-to-lymphocyte ratio, systemic immune-inflammatory index

## Abstract

**Simple Summary:**

Chronic kidney disease is a progressive disease affecting middle-aged and older cats. Recent research has linked chronic inflammatory processes to disease progression. Hemogram-derived inflammatory markers have been studied in many pathological conditions; however, their diagnostic value in feline chronic kidney disease is unknown. The aim of the present study was to investigate selected hemogram-derived inflammatory markers in cats at different clinical stages of chronic kidney disease. The results showed that the values of all the studied inflammatory markers were greater in cats with advanced chronic kidney disease than in healthy cats and in cats with early disease. These findings indicate that cats with advanced chronic kidney disease are in a state of chronic systemic inflammation, which is detectable by simple hematological testing. However, the prognostic value of hemogram-derived inflammatory markers should be assessed in further studies.

**Abstract:**

Chronic kidney disease (CKD) is characterized by chronic inflammation, which mediates the progressive replacement of functional nephrons by fibrotic tissue. Hemogram-derived inflammatory markers are known to serve as markers of pathological conditions; however, their diagnostic value in feline CKD is still unknown. The aim of this retrospective study was to investigate selected hemogram-derived inflammatory markers (neutrophil-to-lymphocyte ratio (NLR), monocyte-to-lymphocyte ratio (MLR), platelet-to-lymphocyte ratio (PLR) and the systemic immune-inflammatory index (SII)) in cats at different clinical stages of CKD. Eighty-eight client-owned cats with CKD and thirty-two healthy control cats were included. Cats with CKD were divided into two groups: early CKD (IRIS stage 1 and 2; 62 cats) and progressed CKD (IRIS stage 3 and 4; 26 cats). The values of inflammatory markers were compared between the two CKD groups and the control group. All investigated hemogram-derived inflammatory markers were significantly (*p* < 0.05) greater in cats with advanced CKD than in those in the other two groups. Additionally, we demonstrated a statistically significant weak to moderate correlation between serum urea, creatinine, selected hematologic and urinary parameters, and the investigated inflammatory markers in cats with CKD. Chronic inflammation can be easily and inexpensively assessed with hemogram-derived markers.

## 1. Introduction

Chronic kidney disease (CKD) is one of the most common causes of mortality in older cats [1,2,3,4,5,6]. However, the etiology and exact pathophysiology are still unclear [7]. CKD is characterized by chronic inflammatory processes in which functional nephrons are progressively lost and continuously replaced by connective tissue. Fibrotic lesions are found in the glomerular, tubular, interstitial, and vascular compartments [8]. In contrast to dogs, interstitial fibrosis, interstitial inflammation, and tubular degeneration predominate over glomerulosclerosis [7,9,10]. The severity of these changes may be linked to the clinical stage of the disease [9,10].

It has also been proposed that chronic inflammatory processes lead to the development and progression of renal fibrosis, but the role of induced myofibroblasts is unclear [8]. The transcript levels of profibrotic genes were found to be positively correlated with creatinine and the severity of the histologic score [11]. Chronic inflammation might lead to the overproduction of superoxide radicals by activated neutrophils (oxidative neutrophil burst) [12]. It has been reported that oxidative burst, expressed as mean fluorescence intensity, produced by optimal neutrophils is significantly greater in cats with stable CKD than in healthy cats [13].

Hemogram-derived inflammatory markers, such as the monocyte-to-lymphocyte ratio (MLR), platelet-to-lymphocyte ratio (PLR), neutrophil-to-lymphocyte ratio (NLR), and systemic immune-inflammatory index (SII), are newly popular, inexpensive, and easily accessible markers of systemic inflammation derived from routine hemogram tests. In human medicine, hemogram-derived inflammatory markers have been described as prognostic and/or diagnostic markers for cancer and inflammatory diseases [14]. There are several reports on the use of hemogram-derived inflammatory markers in veterinary medicine, especially in canine oncology. These markers are recognized as diagnostic and prognostic markers for dogs with lymphomas, osteosarcomas, and mast cell tumors [15]. In cats, the use of hemogram-derived inflammatory markers has been reported with no inflammation, hypertrophic cardiomyopathy, feline mammary carcinomas, acute pancreatitis, feline injection site sarcomas, or high-grade lymphomas [14,15,16,17,18,19].

In human patients with CKD, a significant positive correlation between the PLR and the concentration of high-sensitivity C-reactive protein has been reported [20]. Furthermore, elevated MLR, PLR, and NLR were found to have predictive value for mortality in end-stage human CKD patients [21]. The systemic immune-inflammatory index (SII) was also found to be correlated with prognosis in human CKD patients [22].

However, data regarding hemogram-derived inflammatory markers in feline CKD patients are scarce. A recent study [23] reported increased NLR in cats with CKD at the end of life, which was proposed to be a consequence of the inflammatory whole-body response. Donato et al. [19] reported a weak positive correlation between the NLR and MLR with serum amyloid A and globulin in healthy and diseased cats. However, cats with CKD were not included in this study. Furthermore, the same authors reported a moderate negative correlation between the NLR and the serum albumin concentration and between the NLR and the albumin-to-globulin ratio (AGR), while the correlation between the MLR and the serum albumin concentration and AGR was weak. However, to the best of the authors’ knowledge, hemogram-derived inflammatory markers have not yet been evaluated in cats at different stages of CKD. Therefore, the aim of the present study was to investigate selected hemogram-derived inflammatory markers (NLR, PLR, MLR, and SII) in cats at different clinical stages of CKD.

## 2. Materials and Methods

In this retrospective study, data from hematological, biochemical, and urine analyses of client-owned cats examined from 2010 to 2018 at the Clinic for Small Animals of the Veterinary Faculty in Ljubljana, Slovenia, were analyzed. The cats were included in previously published studies involving research on antioxidant parameters in cats with IRIS stages 1–4 CKD [24] and the effect of diet on the progression of CKD [25].

The inclusion criterion for cats with CKD was a diagnosis of CKD and staging performed according to the International Renal Interest Society (IRIS) guidelines [26]. In order to exclude any possible treatment bias, only cats with no previous treatment were included. In the included cats, urinalysis and ultrasonographic examination were performed as part of routine clinical staging of CKD at presentation.

Cats with acute kidney injury, prerenal or postrenal azotemia, nephropathy of toxic or infectious origin within the last 28 days, urinary tract obstruction, acute systemic inflammation, liver disease, chronic heart failure, or cancer, or cats serologically positive for feline leukemia virus (FeLV) or antibodies against feline immunodeficiency virus (FIV), were excluded. In the case of recorded pyuria, positive urine culture from a sample obtained by cystocentesis, and/or ultrasonographic findings indicative of pyelonephritis, cats were excluded from the present study.

Cats with CKD were divided into two groups: cats with early CKD (IRIS 1 and 2) and cats with progressed CKD (IRIS 3 and 4).

A control group of 32 healthy cats was included, for which the same indexes were calculated. In these cats, the hematological and biochemical analyses and urinalysis were performed either as a part of a wellness checkup or before elective spaying/neutering, and the results were used with the permission of the owners. To be considered healthy, the recorded physical examination, and the results of hematological and serum biochemical analyses and urinalysis, had to be within normal limits and the cats had to be free of FeLV/FIV.

The Committee for Animal Welfare of Veterinary Faculty, University of Ljubljana, Slovenia, and the Ethical Committee of the Ministry of Agriculture, Forestry and Food, Veterinary Administration of the Republic of Slovenia considered that this type of project does not fall under legislation for the protection of animals used for scientific purposes. Owners of the included animals signed informed consent for participation in the study and for the use of all the obtained data for research purposes.

The results of hematological analyses (complete blood count with white blood cell differential counts), biochemical profiles (serum concentrations of urea, creatinine, total proteins, albumins, total calcium, and inorganic phosphate, and the serum activities of alanine aminotransferase and alkaline phosphatase) and the results of urinalysis (urine dipstick analysis, microscopic examination of urine sediment, urine specific gravity (USG), and urine protein-to-creatinine ratio (UPC)) were retrospectively evaluated, but only red blood cell count (RBC), hemoglobin concentration (HGB), hematocrit (HCT), urea, creatinine, USG, and UPC are reported and included in the statistical analysis in the present study.

In all included cats, biochemical analyses were performed with an automated biochemistry analyzer RX Daytona (Randox, Crumlin, UK), and hematological analyses were performed with an automated laser hematology analyzer ADVIA 120 (Siemens, Munich, Germany) using species-specific software (software version 9.9.0-MS). In the case of reported platelet clump flags (PLT-CLM flags in the ADVIA 120 report), the platelet counts were not considered relevant, and the cats were excluded from the study. ADVIA PLT-CLM indicates that more than 1000 platelet clumps were detected in the Perox cytogram.

Furthermore, detection of the FeLV p27 antigen and specific antibodies to FIV was performed using the SNAP FIV/FeLV combo test (IDEXX, Westbrook, ME, USA).

Hemogram-derived inflammatory markers were calculated from the differential white blood cell counts and platelet counts: NLR, the ratio of neutrophil granulocyte concentration (N) to lymphocyte concentration (L); PLR, the ratio of the platelet concentration (P) to L; and MLR, the ratio of the monocyte concentration (M) to L. The systemic immune-inflammation index was calculated as N × P/L.

### Statistical Analysis

Statistical analysis was performed using the SPSS computer program (IBM SPSS 28, Chicago, IL, USA). The Shapiro–Wilk test was performed to determine the distribution of the data. Descriptive statistics were used to describe the demographic and laboratory characteristics of the three groups of cats.

The results of hemogram-derived inflammatory markers, as well as age, weight, concentrations of urea and creatinine, USG, and UPC were not normally distributed and were therefore compared among groups of cats using a nonparametric Kruskal–Wallis test followed by multiple comparisons and Bonferroni adjustments. Red blood cell count, HGB, and HCT were normally distributed and were therefore compared among groups of cats using a parametric one-way ANOVA with post hoc Tukey HSD test. Normally distributed data are reported as mean ± standard deviation (SD) and minimum and maximum values, while non-normally distributed data are reported as medians, minimum and maximum values, and interquartile ranges (25th to 75th percentiles). 

Due to significant age differences between the control group and groups of CKD cats, we performed one-way analysis of covariance (one-way ANCOVA) for normally distributed data and non-parametric ANCOVA (Quade’s test) for non-normally distributed data, with age as the covariate. 

Spearman rank order correlation was used to measure the strength of the correlation between the hemogram-derived inflammatory markers, NLR, MLR, PLR, and SII, and serum urea, serum creatinine, RBC, HGB, HCT, USG, and UPC. The results are presented as *p* values and Spearman’s rank correlation coefficients (r_S_). The absolute magnitude of the observed correlation r_S_ was interpreted as follows: 0.00–0.10: negligible correlation; 0.10–0.39: weak correlation; 0.40–0.69: moderate correlation; 0.70–0.89: strong correlation; and 0.90–1.00: very strong correlation [27]. A value of *p* < 0.05 was considered significant. 

## 3. Results

Altogether, 120 cats were enrolled in this study: 88 cats with CKD and 32 healthy control cats. The enrolled cats were of different breeds and of both sexes. The demographic and laboratory characteristics are presented in Table 1.

Cats in the control group were significantly younger than cats with CKD. However, there was no significant difference in age between the IRIS 1+2 cats and the IRIS 3+4 cats. The body weights of the cats in the IRIS 3+4 group were significantly lower than those of the cats in the control group and of the cats in the IRIS 1+2 group (Table 1).

**Figure 1:**
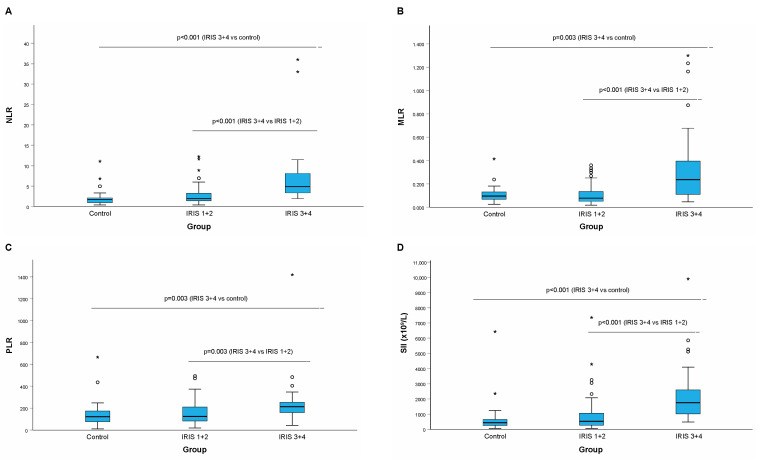
Selected hemogram-derived inflammatory markers ((**A**): NLR; (**B**): MLR; (**C**): PLR, (**D**): SII) in cats with CKD and the control group. Legend: NLR, neutrophil-to-lymphocyte ratio; MLR, monocyte-to-lymphocyte ratio; PLR, platelet-to-lymphocyte ratio; SII, systemic immune-inflammatory index; a small circle (○) is an indication of an outlier; the asterisk (*) is an indication of an extreme outiler.

The levels of all the selected hemogram-derived inflammatory markers were significantly greater in the IRIS 3+4 group than in the IRIS 1+2 and control groups (Table 2, Figure 1A–D). However, there was no difference in the selected hemogram-derived inflammatory markers between the IRIS 1+2 group and the control group. 

**Figure 2:**
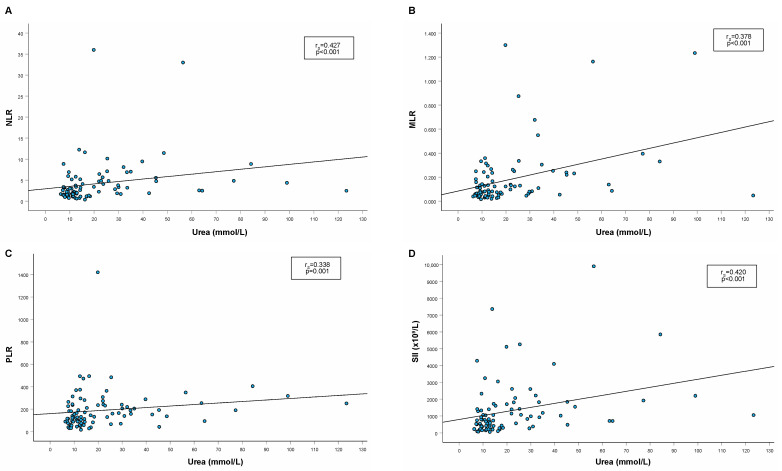
Spearman’s rho correlation between serum urea and hemogram-derived inflammatory markers ((**A**): NLR; (**B**): MLR; (**C**): PLR, (**D**): SII). Legend: NLR, neutrophil-to-lymphocyte ratio; MLR, monocyte-to-lymphocyte ratio; PLR, platelet-to-lymphocyte ratio; SII, systemic immune-inflammatory index; r_S_, Spearman’s rank correlation coefficient.

**Figure 3:**
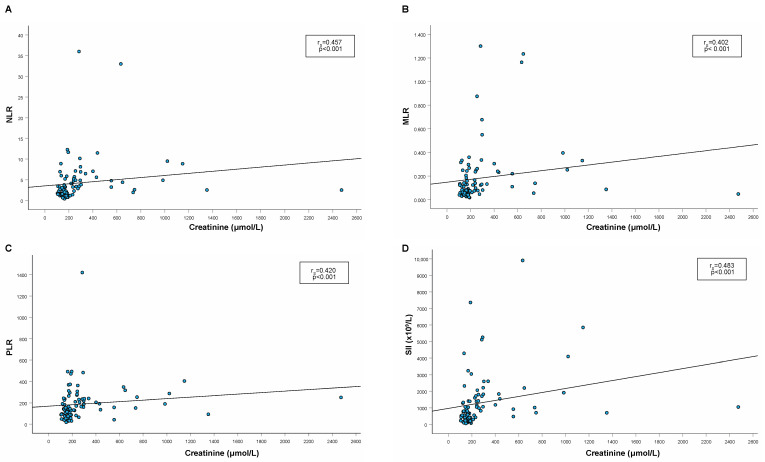
Spearman’s rho correlation between serum creatinine and hemogram-derived inf™∞’lammatory markers ((**A**): NLR; (**B**): MLR; (**C**): PLR, (**D**): SII). Legend: NLR, neutrophil-to-lymphocyte ratio; MLR, monocyte-to-lymphocyte ratio; PLR, platelet-to-lymphocyte ratio; SII, systemic immune-inflammatory index; r_S_, Spearman’s rank correlation coefficient.

In cats with CKD, a significant weak to moderate positive correlation between both serum urea (Figure 2) and creatinine (Figure 3) and all examined hemogram-derived inflammatory markers was found. On the other hand, there was no significant correlation between these parameters in healthy cats.

Furthermore, in cats with CKD, a significant weak to moderate negative correlation between all examined hemogram-derived inflammatory markers and selected hematologic parameters (RBC, HGB, HCT) and USG was found (Table 3). On the other hand, we found a significant weak to moderate positive correlation between all examined hemogram-derived inflammatory markers and UPC. In healthy cats, there was no significant correlation between all selected hematologic parameters and all hemogram-derived inflammatory markers, but USG was significantly negatively correlated with MLR (r_s_ = −0.724, *p* = 0.042), and UPC was significantly positively correlated with NLR (r_s_ = 0.683, *p* = 0.042). Other correlations between USG and UPC and hemogram-derived inflammatory markers were not significant.

The alterations in white blood cell differential count and platelet count in cats with CKD are presented in Table 4. The most common alteration was lymphopenia, which was diagnosed in the majority of cats with advanced CKD and in half of the cats with early CKD.

## 4. Discussion

Our study demonstrated that cats with advanced CKD have significantly greater values of selected hemogram-derived inflammatory markers, NLR, MLR, PLR, and SII, than cats with early CKD and healthy cats. We also demonstrated significant positive correlation between the magnitude of uremia and these inflammatory markers. Furthermore, significant negative correlations were also found between the hemogram-derived inflammatory markers and selected hematologic parameters (RBC, HGB, HCT). Hemogram-derived inflammatory markers significantly negatively correlated with USG and significantly positively correlated with UPC. These results indicate the presence of chronic systemic inflammation in feline CKD patients, which increases with the stage of the disease. To the best of the authors’ knowledge, this is the first report of hemogram-derived inflammatory markers in different stages of feline CKD.

Hemogram-derived inflammatory markers, such as NLR, MLR, PLR, and SII, have been identified as diagnostic and/or prognostic markers for various neoplastic and inflammatory diseases in cats [14,15,17,19,28], but their role in feline CKD has not yet been studied. In human CKD patients, increased values of hemogram-derived inflammatory markers were found to have predictive value for mortality in end-stage CKD patients [21,29,30,31,32,33,34,35]. Furthermore, the SII is recognized as an independent prognostic factor for CKD in human patients [22]. A higher NLR than in healthy cats was already found in cats with CKD at the end of life [23].

In our study, the median NLR and MLR in healthy cats were in accordance with the literature [16,19], and the median PLR was slightly greater than that in previously published data [19]. Cats with advanced CKD had significantly greater levels of all investigated hemogram-derived inflammatory markers than did cats with early CKD and those in the control group.

High NLR, PLR, MLR, and SII values were primarily due to lymphopenia rather than neutrophilia, monocytosis, or thrombocytosis. Lymphopenia was present in the majority of CKD cats, with a greater frequency in advanced stages of CKD. In human CKD patients, lymphopenia has been reported to be a consequence of accelerated apoptosis (premature ageing) of lymphocytes [36,37] and a low relative lymphocyte count has been reported to be independently associated with CKD progression [38]. The lower number of lymphocytes in cats with CKD has already been shown to be negatively correlated with serum creatinine, phosphorus, and urea, leading to a reduced immune response [39]. This could explain why there was a greater frequency of lymphopenia in cats with advanced CKD (73.1%) than in cats with early CKD (50%). The number of lymphocytes in cats with CKD has already been reported to be negatively correlated with serum creatinine, phosphorus, and urea [39]. 

In our study, in cats with CKD but not in healthy cats, there was a weak to moderate positive correlation between serum urea and creatinine and all inflammatory markers, indicating increasing systemic inflammation while the disease worsened. The urinary protein-to-creatinine ratio, independent prognostic factors for worsening of CKD, and USG were also found to be correlated with the hemogram-derived inflammatory markers. The loss of the ability to concentrate urine and proteinuria may appear already in non-uremic cats early in the course of the disease [26]. It was reported that the severity of inflammation measured as the renal interstitial fibrosis score on histopathological examination was significantly correlated with the severity of proteinuria [9]. Hemogram-derived inflammatory markers may therefore be a useful tool in discovering/assessing the state of chronic inflammation in patients early in the course of the disease as well as at its later stages. Furthermore, the selected hematological parameters negatively correlated with the hemogram-derived inflammatory markers. As expected, anemia worsens with the progression of uremia and chronic inflammation. 

The neutrophil-to-lymphocyte ratio is the most widely used hemogram-derived inflammatory parameter and reflects the intensity of neuroendocrine stress and the immune-inflammatory response [40]. Besides lymphopenia, high NLR values could also be a consequence of neutrophilia. Like in human patients [38,41,42], leukocytosis is an independent marker for the progression of feline CKD [43]. Compared with those in healthy cats, significantly greater neutrophil counts and lower lymphocyte counts have been reported in cats with end-stage CKD [39]. However, in our study, neutrophilia was observed in only 26.9% of cats with advanced CKD, and these cats had a significantly greater NLR than did cats with early CKD. On the other hand, it was also reported that in elderly human patients, neutropenia may also be a marker of CKD progression [44]. This was not the case in the present study, where more neutropenic cats were found in the group with early CKD than in the group with advanced CKD.

In addition to lymphopenia, monocytosis could also be a reason for high MLR values. Although cats with advanced CKD had a significantly greater MLR than did cats with early CKD and healthy cats, monocytosis was observed in only two cats with advanced CKD. Monocytosis may occur any time neutrophilia occurs because of a common bipotential stem cell [45]. This may also be caused by the mobilization of marginated cells within the blood vasculature because of elevated endogenic or exogenic cortisol levels; however, this occurs infrequently in cats [45]. Monocytosis in the two cats in our study was accompanied by neutrophilia and was therefore probably caused by an inflammatory response.

Similar to NLR and MLR, both lymphopenia and thrombocytosis could contribute to high PLR values. In our study, only a small percentage of cats with advanced CKD exhibited mild thrombocytosis, defined as platelet counts ranging from 600 to 800 × 10^9^/L [46]. Thrombocytosis may be a result of chronic inflammation [46,47], but blood platelet counts were not reported to be significantly associated with survival in cats with CKD [43]. However, overreactive platelets have been reported in cats with uremic crisis [48]. It is possible that thrombocytosis was not readily observed in the included CKD patients since uremia depresses platelet manufacture in the bone marrow [49]. 

The main limitation of the present study is that there are no known cutoff values for hemogram-derived inflammatory markers in feline CKD patients, which could aid clinicians in better managing feline CKD patients. In human medicine, the cutoff values of hemogram-derived inflammatory markers in different pathologies have already been defined [50,51,52,53,54]. Another limitation is that we could not perform a longitudinal study to evaluate marker dynamics during the progression of the disease. Furthermore, correlation with other commercially used inflammatory markers, such as serum amyloid A and alpha-1-acid glycoprotein, was not possible.

The elevated hemogram-derived inflammatory markers may not be exclusively associated with inflammation but can also occur in conjunction with a stress leukogram. A blood smear analysis to detect band neutrophils would be indicated but was not possible due to the retrospective nature of the study. The authors suggest that chronic stress and chronic inflammation may have occurred simultaneously in the studied population of cats with CKD. Further studies are required to prove this.

Furthermore, due to the retrospective nature of the study, the blood smears could not be evaluated for platelet aggregates. Although the Advia 120 hematologic analyzer relatively reliably reports flags in the presence of platelet clumps, thrombocytopenia resulting in platelet aggregates may not be completely excluded [55,56]. 

In addition, the automated differential leukocyte counts were not confirmed by blood smear examination. Although this may have led to inaccuracy in the reported results, the hematologic analyzer used in this study is reported to be, in addition to its use in microscopic blood smear examination, a reference method in hematology of small animals [57].

Unfortunately, data on serum symmetric dimethylarginine (SDMA) concentrations were only available for 23 cats with early CKD, so the correlation between serum SDMA and hemogram-derived inflammatory markers was not reported.

Finally, the cats in the control group were not age-matched with the diseased cats. As CKD is a disease of the older cat population, it was very difficult to find adult or older cats without concomitant diseases. On the other hand, in the present study, the cats with CKD IRIS 1+2 were significantly older than the cats in the control group, but the hemogram-derived inflammatory markers of cats with IRIS 1+2 did not differ significantly from those in the control group. In addition, the comparison of parameters between the cat groups by the analysis of covariance (ANCOVA) revealed that all parameters that differed significantly between groups of cats without including age as covariate in the statistical analysis remained significantly different even when age was included as a covariate.

Increased values of hemogram-derived inflammatory markers reflect chronic systemic inflammation in feline CKD patients. Although extensively studied in feline CKD using a diversity of inflammatory biomarkers [8,11,13,58,59,60], the extent of chronic inflammation may also be assessed using hemogram-derived inflammatory markers. These markers can be readily calculated and are inexpensive; thus, they may be used in routine clinical settings rather than solely for research. In fact, hematologic analysis is routinely available to veterinarians and is commonly performed as part of standard clinical examination. Another advantage is the small volume of venous blood required to perform the analysis.

## 5. Conclusions

In our study, cats with advanced CKD had significantly greater values of selected hemogram-derived inflammatory markers, NLR, MLR, PLR, and SII, than did cats with early CKD and healthy cats, which may reflect the state of chronic systemic inflammation in feline CKD patients. Additionally, we demonstrated a significant positive correlation between both the serum urea and creatinine concentrations and UPC, and all four investigated inflammatory markers, in cats with CKD but not in healthy cats. Furthermore, selected hematologic parameters (RBC, HGB, HCT) and USG significantly negatively correlated with all hemogram-derived inflammatory markers in cats with CKD.

Further studies are warranted to determine the cutoff values of hemogram-derived inflammatory markers in feline CKD patients and to investigate their role for monitoring feline CKD patients and as independent prognostic markers. Furthermore, their value in detecting subclinical chronic inflammation in patients whose blood cell count is still within normal limits has yet to be determined. 

## Figures and Tables

**Table 1:** Demographic and laboratory characteristics of patients with CKD and healthy controls. Normally distributed data ^N^ are presented as mean ± standard deviation and minimum and maximum values. Non-normally ^NN^ distributed data are presented as the median, minimum and maximum values, and interquartile range (25th to 75th percentiles).

	Control Group	IRIS 1+2	IRIS 3+4
*n*	32	62 (20 IRIS 1/42 IRIS 2)	26 (13 IRIS 3/13 IRIS 4)
Sex (M/F)	18/14	35/27	9/17
Age ^NN^ (years)	6.5 ^a^ (3.0–19.0) [25th: 4.6–75th: 10.0]	10.0 (4.3–12.5) [25th: 6.0–75th: 14.0]	12.5 (4.9–10.3) [25th: 10.0–75th: 15.8]
Body Weight ^NN^ (kg)	4.68 (2.80–8.00) [25th: 3.8–75th: 5.6]	4.58 (2.72–9.10) [25th: 3.8–75th: 5.9]	3.25 ^b^ (2.00–7.40) [25th: 3.0–75th: 4.3]
Urea ^NN^ (mmol/L)	10.2 (6.4–14.2) [25th: 8.2–75th: 11.1]	11.3 (6.3–30.9) [25th: 9.2–75th: 14.3]	34.3 ^c^ (19.8–34.3) [25th: 25.8–75th: 58.1]
Creatinine ^NN^ (µmol/L)	131.5 ^d^ (90.9–148.1) [25th: 116.2–75th: 135.9]	163.7 ^e^ (104.8–246.2) [25th:137.7–75th: 182.9]	415.4 (254.70–2473.8) [25th: 290.1–75th: 738.8]
RBC ^N^ (×10^12^/L)	8.64 ± 1.53 (4.47–11.48)	8.76 ± 1.46 (5.31–13.13)	6.67 ± 1.51 ^c^ (3.38–9.77)
HGB ^N^ (g/L)	125.6 ± 16.2 (91.0–151.0)	121.9 ± 20.9 (54.0–163.0)	98.8 ± 19.3 ^c^ (67.0–139.0)
HCT ^N^ (L/L)	0.41 ± 0.06 (0.28–0.50)	0.39 ± 0.06 (0.23–0.52)	0.30 ± 0.06 ^c^ (0.15–0.440)
USG	1.067 (1.034–1.080) [25th: 1.060–75th: 1.070] (*n =* 8)	1.050 (1.016–1.082) [25th: 1.032–75th: 1.064] (*n =* 45)	
UPC	0.139 (0.066–0.222) [25th: 0.090–75th: 0.160] (*n =* 9)	0.160 (0.061–17.020) [25th: 0.099–75th: 0.320] (*n =* 45)	0.864 ^c^ (0.143–3.760) [25th: 0.545–75th: 1.164] (*n =* 20)

^a^ Significant difference compared to the IRIS 1+2 (*p* = 0.013) and IRIS 3+4 groups (*p* < 0.001). ^b^ Significant difference compared to the IRIS 1+2 (*p* = 0.001) and control groups (*p* = 0.020). ^c^ Significant difference compared to the IRIS 1+2 (*p* < 0.001) and control groups (*p* < 0.001). ^d^ Significant difference compared to the IRIS 1+2 (*p* < 0.001) and IRIS 3+4 groups (*p* < 0.001). ^e^ Significant difference compared to the IRIS 3+4 group (*p* < 0.001). F, female; M, male; *n*, number of cats; ^N^, normally distributed data; ^NN^, non-normally distributed data; RBC, red blood cell count; HGB, hemoglobin concentration; HCT, hematocrit; USG, urine specific gravity; UPC, urine protein-to-creatinine ratio. USG and UPC results were not available for all cats included in individual groups.

**Table 2:** Selected hemogram-derived inflammatory markers in cats with CKD and the control group. The results are presented as the median, maximum and minimum values, and interquartile range [25th to 75th percentile].

	Control Group	IRIS 1+2	IRIS 3+4
NLR	1.70 (0.42–11.09) [25th: 0.97–75th: 2.18]	1.94 (0.39–11.88) [25th: 1.24–75th: 3.29]	4.87 ^a^ (1.93–36.00) [25th: 3.21–75th: 8.12]
MLR	0.095 (0.026–0.414) [25th: 0.067–75th: 0.132]	0.075 (0.017; 0.359) [25th: 0.0475–75th: 0.136]	0.240 ^b^ (0.046; 1.300) [25th: 0.110–75th: 0.550]
PLR	123.3 (11.3–666.7) [25th: 74.11–75th: 175.9]	124.9 (18.2–494.3) [25th: 80.4–75th: 214.6]	205.6 ^c^ (42.4–1420.0) [25th: 157.7–75th: 253.7]
SII (×10^9^/L)	431.0 (53.4–6433.3) [25th: 261.2–75th: 659.7]	527.9 (60.5–7360.6) [25th: 274.9–75th: 1056.4]	1705.4 ^a^ (477.1–9900.0) [25th: 982.7–75th: 2600.2]

^a^ Significant difference compared to the IRIS 1+2 (*p* < 0.001) and control groups (*p* < 0.001). ^b^ Significant difference compared to the IRIS 1+2 (*p* < 0.001) and control groups (*p* = 0.003). ^c^ Significant difference compared to the IRIS 1+2 (*p* = 0.003) and control groups (*p* = 0.003). NLR, neutrophil-to-lymphocyte ratio; MLR, monocyte-to-lymphocyte ratio; PLR, platelet-to-lymphocyte ratio; SII, systemic immune-inflammatory index.

**Table 3:** Spearman’s rho correlation between hemogram-derived inflammatory markers and selected hematologic and urine parameters.

	NLR	MLR	PLR	SII (×10^9^/L)
RBC (×10^12^/L) (*n* = 88)	r_S_ = −0.553 *p* < 0.001	r_S_ = −0.396 *p* < 0.001	r_S_ = −0.458 *p* < 0.001	r_S_ = −0.600 *p* < 0.001
HGB (g/L) (*n =* 88)	r_S_ = −0.436 *p* < 0.001	r_S_ = −0.286 *p* = 0.007	r_S_ = −0.354 *p* < 0.001	r_S_ = −0.530 *p* < 0.001
HCT (L/L) (*n =* 88)	r_S_ = −0.554 *p* < 0.001	r_S_ = −0.421 *p* < 0.001	r_S_ = −0.465 *p* < 0.001	r_S_ = −0.637 *p* < 0.001
USG (*n =* 67)	r_S_ = −0.584 *p* < 0.001	r_S_ = −0.462 *p* < 0.001	r_S_ = −0.439 *p* < 0.001	r_S_ = −0.638 *p* < 0.001
UPC (*n =* 65)	r_S_ = 0.641 *p* < 0.001	r_S_ = 0.549 *p* < 0.001	r_S_ = 0.451 *p* < 0.001	r_S_ = 0.661 *p* < 0.001

r_S_, Spearman’s rank correlation coefficient; NLR, neutrophil-to-lymphocyte ratio; MLR, monocyte-to-lymphocyte ratio; PLR, platelet-to-lymphocyte ratio; SII, systemic immune-inflammatory index; RBC, red blood cell count; HGB, hemoglobin concentration; HCT, hematocrit; USG, urine specific gravity; UPC, urine protein-to-creatinine ratio.

**Table 4:** Frequency of alterations in hematological parameters in cats with CKD.

Change in Hemogram	IRIS 1+2 (*n =* 62)	IRIS 3+4 (*n =* 26)
Leukopenia (WBC < 6.3 × 10^9^/L)	19 (30.6%)	2 (7.7%)
Lymphopenia (L < 2.0 × 10^9^/L)	31 (50%)	19 (73.1%)
Neutrophilia (N > 13.4 × 10^9^/L)	2 (3.2%)	7 (26.9%)
Neutropenia (N < 3.0 × 10^9^/L)	13 (21.0%)	2 (7.7%)
Monocytosis (M > 1.0 × 10^9^/L)	0 (0%)	2 (7.7%)
Thrombocytosis (P > 626.4 × 10^9^/L)	0 (0%)	2 (7.7%)
Thrombocytopenia (P < 156.4 × 10^9^/L)	11 (17.7%)	3 (11.5%)

L, lymphocyte concentration; M, monocyte concentration; n, number of cats; N, neutrophil granulocyte concentration; P, platelet concentration; WBC, white blood cell count.

## Data Availability

The data presented in this study are available on: http://hdl.handle.net/20.500.12556/RUL-158608.
